# Massive Rectal Hemorrhage From a Stercoral Ulcer

**DOI:** 10.7759/cureus.26963

**Published:** 2022-07-18

**Authors:** Samara Hassranah, Sandeep Maharajh, Sanjeev Solomon, Vijay Naraynsingh

**Affiliations:** 1 Surgery, Medical Associates Hospital, St. Joseph, TTO; 2 Gastroenterology, Medical Associates Hospital, St. Joseph, TTO; 3 Clinical Surgical Sciences, The University of the West Indies, St. Augustine, TTO

**Keywords:** colonoscopy, colectomy, rectal bleed, massive gastrointestinal bleed, stercoral ulcer

## Abstract

Massive lower gastrointestinal (GI) bleeding from stercoral ulcers is exceedingly rare. We report a case of a middle-aged man who presented with progressively deteriorating neurologic function with constipation and subsequent massive GI bleeding per rectum. While an uncommon cause of GI bleeding, such patients require rapid resuscitation and timely diagnosis of these ulcers since the usual management of such cases will be futile and harmful due to potentially inappropriate surgical bowel resection.

## Introduction

Stercoral ulceration is a rare complication of constipation where stool impaction causes pressure necrosis of the bowel wall [1]. First described by Berry in 1894, there has since been little literature describing the phenomena; approximately 150 cases were reported by 2016 [2,3]. The incidence is unknown among the general population, and massive bleeding is an exceedingly rare complication, sometimes being described as “relatively unknown” [1,4,5]. To add to the available data, we report our experience with life-threatening bleeding from a stercoral ulcer.

## Case presentation

A 50-year-old man, with no medical history, reported one month of worsening neurological deficits after the onset of neck pain. He also had retention of urine and constipation. Progressive numbness and weakness in his lower limbs, combined with MRI findings, revealed a diagnosis of transverse myelitis. He was catheterized, and the constipation was managed with lactulose.

**Figure 1:**
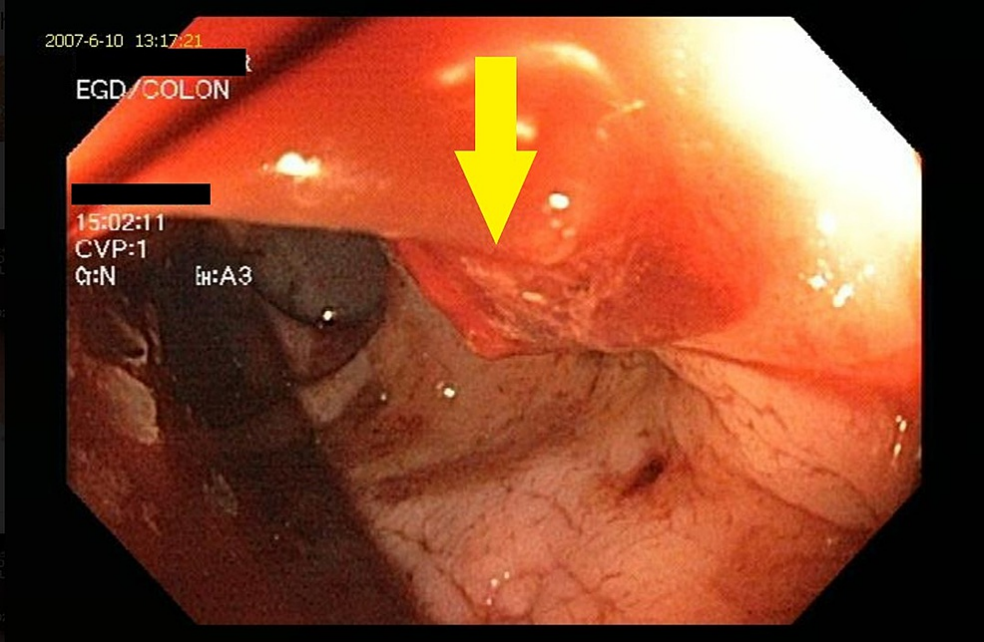
Colonoscopy showing linear oozing rectal stercoral ulcer (yellow arrow).

After three weeks, he had three episodes of bright red per rectal bleeding that progressively worsened, and he went into hemorrhagic shock. After resuscitation, emergency colonoscopy showed a blood-filled rectum with a 7mm actively bleeding linear ulcer 3cm from the anorectal junction (Figure 1). From the sigmoid colon proximally, there was brown stool and no blood. Hemostasis was achieved with epinephrine injections and hemostatic clips to the ulcer, and once stable, the patient was discharged surgically.

**Figure 2:**
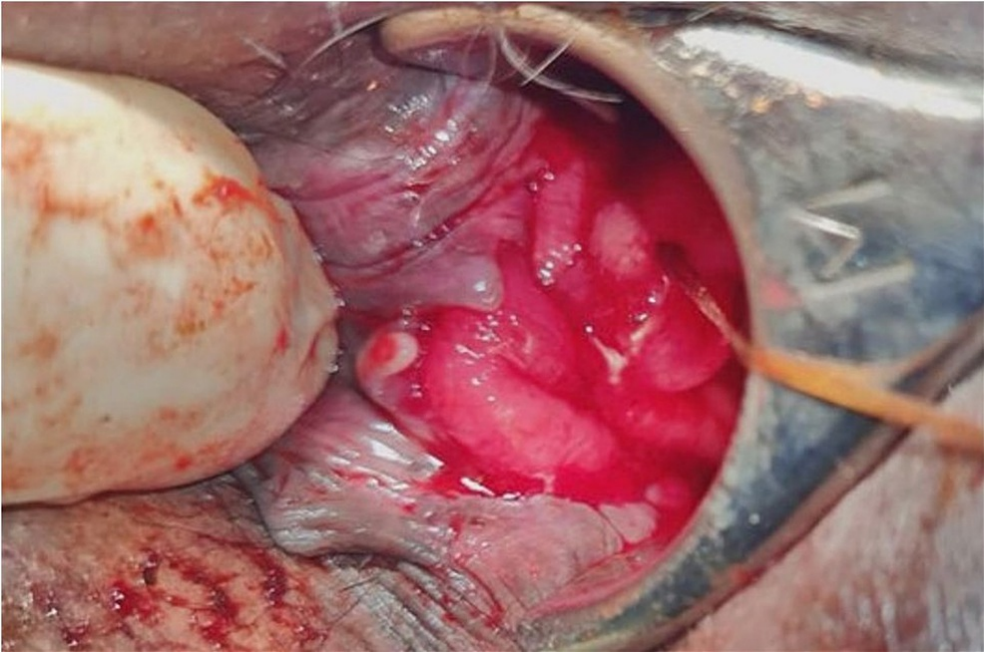
Hill Ferguson retractor allowing visualization for transanal oversewing of the bleeding stercoral ulcer.

Eight days after discharge, he was readmitted for more episodes of massive rectal bleeding. He was resuscitated, and transanal oversewing of the ulcer performed under general anesthesia achieved effective hemostasis (Figure 2).

## Discussion

Diverticulosis and angiodysplasia are the two commonest causes of massive lower gastrointestinal (GI) bleeding; these account for 50% and 12% of presentations, respectively, but 10-15% are upper GI bleeds [6-8]. After resuscitation, urgent endoscopy can usually identify and treat the lesion [9]. Angiography can also locate bleeding, but the rate must be more than 0.5ml/minute [10]. Surgery is indicated when the patient is unstable hemodynamically or if there is transfusion with more than 6 units of blood in 24 hours or failure of endoscopy; if the bleeding site has not been identified, subtotal colectomy is the recommended treatment [11]. In patients with constipation and rectal bleeding, stercoral ulcer must be considered as a source of bleeding since blind subtotal colectomy for rectal bleeding can result in increased morbidity and mortality.

Colonoscopy for rectal bleed diagnosed the ulcers in studies by Sugawa et al. and Komai, and emergency colonoscopy diagnosed them in case reports by Huang et al., Matsushita et al., and Madan et al. [1,5,12-14].

Stercoral ulcers are uncommon. Maurer et al. determined that they only accounted for 3.2% of all colonic perforations, and Sugawa et al. found only 14 cases when 54 cases of rectal ulcers were reviewed [12,15].

There is little in the literature on hemorrhaging stercoral ulcers accounting for the limited data on death rates [16]. Case reports by Knigge and Katon and Matsushita et al. documented successful interventions for patients who had rectal bleeding, while Madan et al. recorded a case where bleeding stopped spontaneously. Milliser et al. and Kato et al. documented cases where the patients did not survive [4,5,14,17,18].

Treatments for bleeding stercoral ulcers, and rectal ulcers in general, differ from other causes of painless lower GI bleed. Management of bleeding rectal ulcers with endoscopic epinephrine injections was described by Sugawa in four patients, and in two of them, the heater probe was also used as part of the management, but treatment of bleeding stercoral ulcers, specifically, has limited documentation [12]. Knigge et al. reported endoscopic multipolar coagulation and injection therapy that successfully treated a stercoral ulcer, while Huang et al’s and Matsushita et al’s case reports discuss using injection therapy with epinephrine alone in the management of their patients’ ulcers [1,4,14]. However, bleeds that are refractory to these treatments need to be managed surgically. In 1995, Binderow et al. described abdominal rectosigmoid resection for two patients in whom bleeding from a solitary rectal ulcer was otherwise uncontrollable, but these patients did not survive [19]. It would appear therefore that transanal control of hemorrhage by oversewing the ulcer may be preferable to a major abdominopelvic resection in very ill patients.

Though rare, it is imperative to remember this condition when encountered with a patient who has chronic constipation and presents with hematochezia. Subtotal colectomy cannot be used to manage bleeding stercoral ulcerations as they are usually located at or distal to the rectosigmoid junction [20]; if done, patients will continue to have massive hemorrhage and will have undergone an operation that will likely do more harm. Colonoscopy for diagnosis before surgery is ideal and ensures that the traditionally appropriate management of subtotal colectomy for massive rectal bleed is not performed inappropriately. This report of successful hemostasis with surgical intervention begins to fill the need for more documentation of techniques for bleeding stercoral ulcers.

## Conclusions

Massive per rectal bleeding is a common surgical emergency. Patients can be hemodynamically unstable, and timely resuscitation and diagnosis of etiology is the cornerstone of management. While subtotal colectomy has been the traditional management of this emergent surgical condition, it will not address rectal pathology. Therefore, colonoscopy and recto-anal assessment is crucial in the emergent management of patients with massive per rectal bleeding, especially in those with a chronic history of constipation in which bleeding stercoral ulcers can be present.
